# Case Report: A complicated case of neonatal refeeding syndrome following intrauterine growth restriction corrected by high dose thiamine supplementation

**DOI:** 10.3389/fped.2025.1594715

**Published:** 2025-07-31

**Authors:** Yixiang Wu, Hongxun Xu, Lizhong Du, Zhongyue Li

**Affiliations:** ^1^ Department of Pediatric, The Fourth Affiliated Hospital, Zhejiang University School of Medicine, Yiwu, Zhejiang, China; ^2^ Department of Pediatric, Children’s Hospital Zhejiang University School of Medicine, Hangzhou, Zhejiang, China

**Keywords:** refeeding syndrome, electrolyte imbalance, thiamine, intrauterine growth restriction, case report

## Abstract

Refeeding syndrome (RS), marked by severe electrolyte imbalances (e.g., hypophosphatemia, hypokalemia) and thiamine deficiency, poses significant risks during nutritional rehabilitation in intrauterine growth restriction (IUGR) neonates. This case report highlights the efficacy of high-dose thiamine (2 mg/kg IV) in resolving refractory RS in a preterm IUGR infant (34 weeks, 1,415 g) unresponsive to standard electrolyte correction. Despite gradual caloric reintroduction and parenteral supplementation, the infant exhibited persistent hypophosphatemia (3.0 mmol/kg/day IV requirement), hypokalemia (4.3 mmol/kg/day IV requirement), and thrombocytopenia (nadir 27 × 10^9^/L). Thiamine administration led to rapid clinical improvement within 4 h, with electrolyte normalization (potassium: 2.19→4.41 mmol/L; phosphorus: 0.69 →2.35 mmol/L) and platelet recovery (27→112 × 10^9^/L). The findings suggest thiamine deficiency may underlie refractory RS in IUGR neonates, advocating for early supplementation in high-risk cases. Further research is needed to optimize dosing and validate thiamine's role in RS management.

## Introduction

Refeeding syndrome (RS), characterized by life-threatening electrolyte imbalances (e.g., hypophosphatemia, hypokalemia) and thiamine deficiency, is a critical complication during nutritional rehabilitation in neonates with intrauterine growth restriction (IUGR) (1–3). Despite ASPEN guidelines recommending gradual caloric reintroduction, refractory RS remains challenging in preterm IUGR infants due to severe placental insufficiency and metabolic vulnerability (4–6). Current protocols emphasize electrolyte correction, yet thiamine's role in reversing refractory RS is understudied. This case highlights the efficacy of high-dose thiamine (2 mg/kg) in resolving persistent RS manifestations.

## Case report

**Figure 1:**
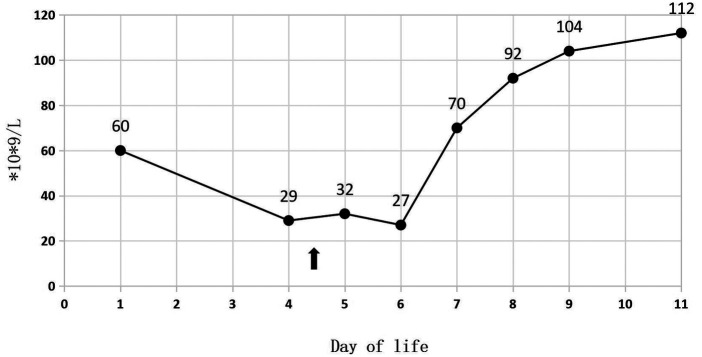
Platelet during 11 days of life.

A 34-week-old female premature infant, weighing 1,415 g (at the 3rd percentile on the female fetal growth curve), was delivered via cesarean section to a 23-year-old mother, G2P1, due to diminished fetal activity. The mother's pre-pregnancy body mass index (BMI) was 29.4, and she experienced a weight gain of 20 kg throughout the duration of her pregnancy. Her dietary intake was generally consistent with typical eating patterns. And she has a history of delivering a full-term baby girl weighing 2,500 g at birth. Following the prenatal diagnosis of severe IUGR and oligohydramnios, the baby was admitted for potential delivery. Her sibling is in good health, and the parents are not consanguineous. The mother received the full course of antenatal dexamethasone. Following birth, the Apgar scores were recorded as 7, 8, and 9. The patient was subsequently admitted to NICU after undergoing initial resuscitation and receiving blended oxygen via a nasal cannula. Blood glucose level was 1.7 mmol/L at the age of 0.5 h after NICU admission. Intravenous glucose fluid was administered. By the first day of life, the infant's vital signs remained stable, and total parenteral nutrition (TPN) was initiated at 16.5 h. On the second day, trophic feeding was initiated. The initial CBC revealed a total white blood cell count of 6.2 × 10^9^/L; while the absolute neutrophil count (ANC) was 2.8 × 10^9^/L. Her platelet count at birth was 60 × 10^9^/L (Figure 1), and hemoglobin measured at 18.8 g/dl.

**Figure 2:**
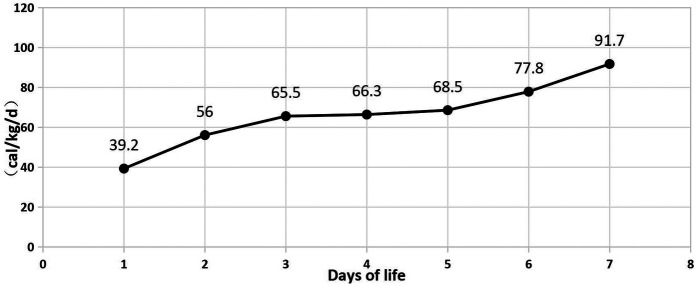
Calorie during 7 days of life.

**Figure 3:**
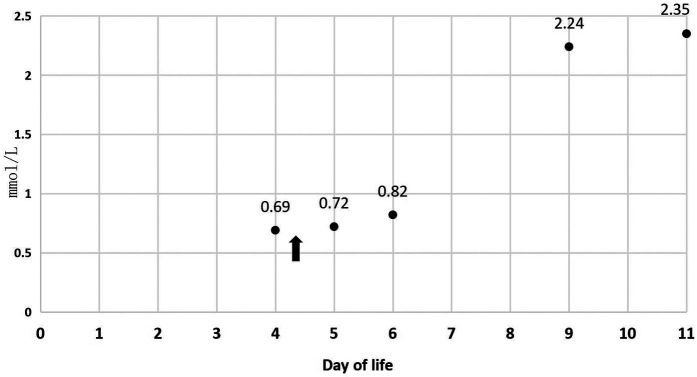
P during 11 days of life.

**Figure 4:**
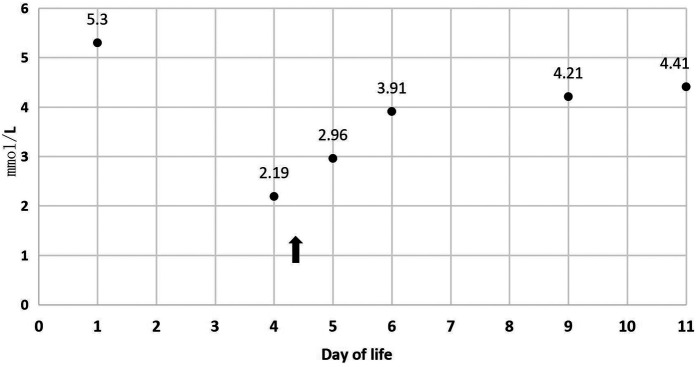
K^+^ during 11 days of life.

In Table 1, the glucose infusion rate (GIR) is presented, as well as the quantities of proteins and lipids supplied within the initial week of TPN, alongside the daily caloric intake (Figure 2). Enteral feeding could not be increased due to increased gastric residuals and abdominal distension, resulting in the patient's dependence on TPN. The TPN protocol in our unit stipulates that both water and lipid-soluble vitamins should be started after birth, and the patient was provided with supplemented TPN. The routine vitamins in the TPN have maintained thiamine with a dose of 0.40 mg/kg since TPN was initiated on day one of life. As the patient's intravenous nutrition was delivered via peripheral veins and that the calcium ion levels were within the normal elevated range (Table 2), calcium supplements were not incorporated into the intravenous nutrition solution. Electrolyte imbalance, including hypokalemia and hypophosphatemia, was noted after the third day of life. At DOL 3, IV potassium requirement reached 4.3 mmol/kg/day and IV phosphate requirement reached 3.0 mmol/kg/day. However, hypokalemia and hypophosphatemia were refractory to correction with intravenous (IV) supplementation. At the same time, the patient had progressive thrombocytopenia, which reached its nadir of 27 × 10^9^/L at DOL 4. Furthermore, severe thrombocytopenia was unresponsive to high doses of intravenous immunoglobulin. During the NICU stay, there was no occurrence of patent ductus arteriosus or intracranial hemorrhage. TORCH testing for congenital infections was negative. Due to the disturbances in electrolytes and the presence of thrombocytopenia, there was a suspicion of refeeding syndrome accompanied by thiamine deficiency. Thiamine levels were not measured due to the unavailability of the test at our hospital. A significant improvement in the infant's condition was observed four hours following the intravenous administration of thiamine at a dosage of 2 mg/kg on day of life four. Along with this, the imbalance in electrolytes and thrombocytopenia were corrected (Figures 1, 3, 4). According to the 2020 ASPEN guidelines for RS, thiamine replacement therapy was maintained at a dose of 2 mg/kg/day IV for an additional two consecutive days. Afterward, a maintenance dose of 0.4 mg/kg/day was given each day using TPN. Following that, the infant remained stable with no additional disturbances in electrolytes. At one month of age, she was discharged with a corrected gestational age of 38 weeks and 4 days, and a weight of 2.16 kg, which is approximately at the 3rd percentile of the growth curve. The child's parents consented to the department's treatment and, after discharge, adhered to the doctor's recommendation to use breast milk fortifiers for enhanced nutrition and electrolyte balance. By the corrected gestational age of 42 weeks, with full-dose fortified breast milk, her weight reached 3.5 kg, around the 30th percentile on the growth chart.

**Table 1:** Total parenteral nutrition, glucose infusion rate and daily total caloric intake during the first week of life.

Nutrients	Day of life						
	1	2	3	4	5	6	7
GIR (g/kg/d)	9.8	8.8	7.3	7.9	8.2	9.5	10.5
Proteins (g/kg/d)	0	2	2.7	3.5	3.5	3.4	3.3
Lipids (g/kg/d)	0	1	2	2.3	2.0	2.5	2.8
K+ (mmol/kg/d)	0	0	0	4.3	4.0	2.0	2.0
P (mmol/kg/d)	0	1.0	1.0	3.0	3.0	2.0	2.0
Total calorie (cal/kg/d)	39.2	56	65.5	66.3	68.5	77.8	91.7

**Table 2:** Electrolyte, lactic acid and blood glucose during 11 days of life.

Metabolic and electrolyte profile	Day of life					
	1	4	5	6	9	11
Total serum Ca (mmol/L)	/	1.93	2.36	2.54	2.32	2.37
Ionized Ca (mmol/L)	1.35	1.2	1.25	/	1.34	1.32
P (mmol/L)	/	0.69	0.72	0.82	2.24	2.35
K^+^ (mmol/L)	5.3	2.19	2.96	3.91	4.21	4.41
Mg^2+^ (mmol/L)	/	0.64	0.64	0.86	0.82	0.88
Lactic acid (mmol/L)	5.2	2.2	3.2	/	2.2	2.6
Blood glucose (mmol/L)	1.7–6.2	8.6	7.2	7.0	5.5	5.8

## Discussion

IUGR infants have been exposed to an unfavorable environment *in utero* due to placental insufficiency, a primary factor contributing to fetal undernutrition. Studies have shown that placental dysfunction is responsible for the development of severe postpartum hypophosphatemia (7). While providing early nutritional support with increased calories and protein intake during the first week of life reduces mortality rates and supports neurodevelopment in IUGR infants, it may also be associated with adverse conditions such as acute electrolyte imbalances and late-onset sepsis (8, 9). Since neurodevelopment is crucial in early infancy, implementing positive early nutrition is recommended for IUGR newborns (10–12).

Refeeding syndrome generally emerges during the first few days of active oral or intravenous nutritional support. During the refeeding process, carbohydrate consumption stimulates insulin secretion, which shifts metabolic pathways from catabolic to anabolic states. This transition creates an immediate demand for inorganic phosphate to synthesize adenosine triphosphate (ATP). Additionally, the intracellular transport of glucose necessitates potassium, biosynthetic reactions require magnesium, and the oxidative metabolism of carbohydrates and amino acids is dependent on thiamine (13). This overnutrition could lead to excess usage and intake of phosphate at the cellular level, and result in increased hypophosphatemia (14, 15). At the same time, carbohydrate refeeding is believed to enhance cellular thiamine use since it acts as a cofactor for several enzymatic processes, such as transketolases (16). Thiamine shortage is typically associated with refeeding syndrome (17). When carbohydrates are introduced, metabolism shifts from lipids to carbohydrates, potentially leading to acute thiamine deficiency due to increased carbohydrate metabolism and thiamine use during glycolysis (18). Consequently, the electrolyte disturbances noted in RS cases could result from a movement from the extracellular to the intracellular space.

Thiamine functions as a co-factor during the consumption of glucose aerobically. Thiamine deficiency, also known as beriberi, can lead to intrauterine growth restriction (IUGR) due to its critical role in energy metabolism and cellular processes. Thiamine is an essential vitamin that plays a key role in converting carbohydrates into energy in the body. In pregnant women, thiamine deficiency can lead to a decrease in the amount of energy available to the developing fetus, which can result in growth restriction. Thiamine deficiency can also affect other cellular processes in the body, including DNA synthesis and nerve function. These effects can contribute to the development of IUGR and other complications in pregnancy. In addition to its direct effects on fetal growth and development, thiamine deficiency can also lead to maternal complications such as heart failure, which can further impact fetal growth and well-being. In the absence of adequate thiamine, a dual enzyme defect occurs, causing a disruption in aerobic metabolism and leading to insufficient ATP generation. Additionally, the conversion of pyruvate to lactate causes increased lactate concentration and lactic acidosis. Deficiency in thiamine can affect numerous organ systems due to the presence of thiamine-dependent metabolic pathways in nearly all human cells. Additionally, it can intensify the hypomagnesemia, hypokalemia, and hypophosphatemia linked to greater renal losses. Deficiency in thiamine (vitamin B1) might interfere with the function of renal tubules, resulting in lower electrolyte reabsorption due to compromised ATP-dependent transport systems (19, 20). The dramatic response to a single thiamine dose supports this concept. Through its involvement in transketolase activity, thiamine is essential for protection against oxidative stress (21, 22). Thiamine deficiency can cause acute tubular necrosis (ATN) through ischemia-reperfusion injury, due to inadequate ATP generation and the harmful effects of reactive oxygen species (23). Therefore, timely supplementation of thiamine can restore proximal renal tubular function and alleviate the symptoms of RS.

The mechanism of thrombocytopenia in IUGR is not fully understood, but it is thought to be related to a reduced production of platelets in the bone marrow or increased destruction of platelets. A prospective study conducted at a single center demonstrated that in non-small for gestational age (non-SGA) infants with thrombocytopenia, thrombopoietin levels are elevated to enhance platelet production. Conversely, in small for gestational age (SGA) infants, thrombopoietin production is inadequate, leading to a marked reduction in platelet count (24). IUGR can also result in reduced blood flow to the fetus, leading to hypoxia, oxidative stress, and inflammation, which can contribute to thrombocytopenia. Vitamin B1, also known as thiamine, is essential for the normal functioning of the body's metabolic processes. However, its role in the treatment of thrombocytopenia in IUGR is not well established. Currently, there is limited research on the efficacy of vitamin B1 in treating thrombocytopenia, and further studies are needed to fully understand its effects. In general, the management of thrombocytopenia in IUGR involves addressing the underlying causes and may include blood transfusions, corticosteroids, or other medications to increase platelet production or reduce destruction.

## Conclusions

Placental dysfunction leading to IUGR in preterm infants can result in severe hypophosphatemia and hypokalemia, resembling refeeding syndrome-like metabolic issues. Careful observation of electrolyte levels and modifying the amounts of electrolytes, amino acids, and calories are crucial for managing VLBW preterm infants, particularly those with IUGR. Besides implementing the ESPGHAN 2018 guideline of administering 0.8–2.0 mmol/kg/d IV phosphate on the first day and then 1.6–3.5 mmol/kg/d IV phosphate during the first week, regular monitoring of calcium and phosphate levels in the initial 5 days after birth is also necessary for infants. Particularly for extremely low birth weight (ELBW) infants experiencing refeeding syndrome accompanied by persistent electrolyte abnormalities, an increased dosage of thiamine is advised. However, additional research is needed to determine if aggressive nutrition protocols tailored for preterm infants of appropriate gestational age can be applied to IUGR infants.

## Data Availability

The original contributions presented in the study are included in the article/Supplementary Material, further inquiries can be directed to the corresponding author.
